# Epicardial Ventricular Tachycardia Ablation Guided by a Novel High‐Resolution Contact Mapping System: A Multicenter Study

**DOI:** 10.1161/JAHA.118.010549

**Published:** 2018-10-30

**Authors:** Rui Shi, Zhong Chen, Andrianos Kontogeorgis, Frederic Sacher, Paolo Della Bella, Caterina Bisceglia, Ruairidh Martin, Christian Meyer, Stephan Willems, Vias Markides, Philippe Maury, Tom Wong

**Affiliations:** ^1^ Department of Cardiovascular Medicine The First Affiliated Hospital of Xi'an Jiaotong University Xi'an China; ^2^ Heart Rhythm Centre The Royal Brompton and Harefield NHS Foundation Trust National Heart and Lung Institute Imperial College London United Kingdom; ^3^ Bordeaux University Hospital LIRYC Institute INSERM 1045 Bordeaux University Bordeaux France; ^4^ Arrhythmia Unit and Electrophysiology Laboratories San Raffaele University Hospital Milan Italy; ^5^ Department of Cardiology Electrophysiology cNEP Cardiac Neuro and Electrophysiology research group University Heart Centre University Hospital Hamburg—Eppendorf Hamburg Germany; ^6^ DZHK (German Centre for Cardiovascular Research), partner site Hamburg/Kiel/Lübeck Germany; ^7^ University Hospital Rangueil Toulouse France

**Keywords:** catheter ablation, epicardial, ventricular arrhythmia, Arrhythmias

## Abstract

**Background:**

Mapping using a multipolar catheter with small and closely spaced electrodes has been shown to improve the validity of electrograms to identify endocardial critical sites of reentry isthmus and foci of earliest activation. However, the feasibility, safety, and clinical outcome of using such technology to guide epicardial ventricular tachycardia (VT) ablation has not been reported.

**Methods and Results:**

Thirty‐three consecutive patients from 5 high‐volume centers were studied. These patients had 43 epicardial maps using a novel 64‐pole mini‐basket catheter to guide VT ablation. Activation maps with 17 832 points per map (interquartile range: 7621–32 497 points per map) were acquired in 11 patients with tolerated VT (7 focal, 4 reentry). Substrate maps with 40149 points per map (interquartile range: 20926–49391 points per map) were acquired in 30 patients. Local abnormal ventricular activities were consistently demonstrated at the substrate regions of interest. Epicardial ablation was performed in 31 of 33 patients, with acute VT termination in 10 of 11 patients (91%). Complete elimination of local abnormal ventricular activities was achieved in 25 of 31 patients. At a median follow‐up of 10 months (interquartile range: 4–14 months), 64% (7/11) of patients who had acute termination of VT and 55% (11/20) of those who had substrate modification alone were free of VT. There was no immediate complication following epicardial procedure.

**Conclusions:**

Epicardial VT ablation guided by a mini‐basket catheter is feasible and safe. Complete reentry VT circuits and foci of earliest activation were identified in all inducible stable VT. The longer term clinical outcome of ablation guided by this novel mapping technology utilizing small and closely spaced electrodes will have to be determined with a larger study.

**Clinical Perspective**

**What Is New?**

- This study is the first to report on the clinical utility of a 64‐pole mini‐basket catheter to map epicardial ventricular tachycardia from 5 large‐volume centers.
- In a collapse state, electroanatomical mapping using the mini‐basket catheter is feasible and safe within the confined space of the pericardium.

**What Are the Clinical Implications?**

- High‐density mapping technology is able to identify all reentry ventricular tachycardia circuits, earliest activation foci, and ventricular substrate to guide epicardial ablation treatment of ventricular tachycardia, with favorable immediate and median‐term ablation outcome.
- The longer term clinical outcome from using this rapid, high‐density, and high‐resolution electroanatomical mapping to guide epicardial ablation procedures will have to be pursued in further, larger studies.

## Introduction

The epicardially located critical isthmus, focus, and substrate for ventricular tachycardia (VT) are recognized in patients with nonischemic cardiomyopathy.^1^, ^2^ Increasingly, such epicardial involvement has also been recognized in ischemic cardiomyopathy patients who previously failed endocardial ablations. Between 13% and 17% of VT circuits or foci were found to be epicardially located.^3^, ^4^

In comparison to conventional mapping technology, catheters with smaller and closely spaced electrodes have been shown to be better at identifying viable tissue at the scar border zone or within the scar.^5^ We previously showed that endocardial VT mapping and substrate characteristics using a 64‐pole mini‐basket catheter (IntellaMap Orion; Boston Scientific) with small and closely spaced electrodes is feasible and safe^6^, ^7^; however, its application to mapping the epicardial ventricular surface has not been studied previously.

We report on the initial Europe‐wide experience using this mini‐basket catheter to identify the VT circuits, foci, and substrates to guide epicardial ablation in treating VT, with a particular focus on feasibility, safety, and efficacy.

## Methods

The data, analytic methods, and study materials will not be made available to other researchers for purposes of reproducing the results or replicating the procedure.

### Patients

Patients who underwent epicardial electroanatomical mapping using the 64‐pole mini‐basket catheter paired with the Rhythmia mapping annotation system (Boston Scientific) in 5 high‐volume electrophysiology centers (London, Toulouse, Bordeaux, Milan, Hamburg) between January 2015 and January 2017 for suspected epicardial VT were included in the study. Patients’ clinical and procedural information, including electroanatomical mapping data, was obtained for analysis. All patients gave written informed consent as part of their clinical procedure. The data review for research was approved by the research ethics committee institutional review boards.

### Electrophysiology Procedure

The decision to use an epicardial electroanatomical mapping and ablation approach was made on the basis of the body‐surface 12‐lead ECG morphology, failure of prior endocardial ablation, and/or likelihood of epicardial VT substrate based on cardiac pathology.^8^

A percutaneous subxiphoid puncture approach was used to obtain access to the pericardial space, as described previously.^9^ The mini‐basket catheter, in its collapsed state, was inserted to the pericardial space via a steerable sheath. Substrate mapping was performed during sinus or atrial/ventricular paced rhythm depending on preferred wave‐front directions. Attempts were then made to induce clinical VT. If sustained VT was induced with tolerable hemodynamic status, the VT activation map was then acquired using the mini‐basket catheter epicardially. Radiofrequency ablation was then performed, targeting the critical isthmus or the earliest activation with the aim of achieving VT termination. Further substrate ablation was then performed with the complete elimination of local abnormal ventricular activity (LAVA) including late potentials and noninducibility of clinical VT as the procedure end points.^10^, ^11^ If the clinical VT was not hemodynamically stable or not inducible, then substrate ablation alone was performed in targeting the LAVA.

### Epicardial Electroanatomical Mapping Using a Mini‐Basket Catheter

**Figure 1:**
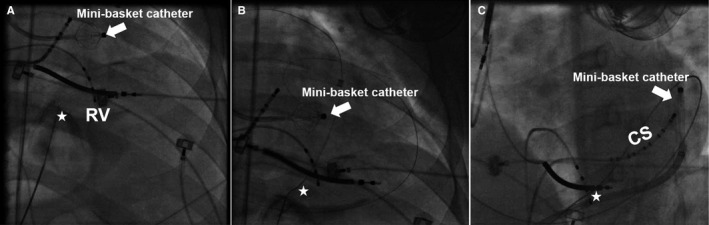
Epicardial access and mapping by a mini‐basket multipolar catheter. The arrow points to the mini‐basket catheter, and the star identifies the point of epicardial access. Pericardial puncture using a micropuncture needle (**A**) and insertion of a guide wire into the epicardial space (**B**) illustrate the percutaneous subxiphoidal puncture approach used to access to the epicardial space. The mini‐basket catheter was fully deployed (balloon shape) endocardially when inside the left ventricle via a transaortic retrograde approach. **C**, The mini‐basket catheter was undeployed (spindle shape) for mapping in the confined epicardial space. CS indicates coronary sinus; RV, right ventricle.

The 64‐pole mini‐basket catheter contained 8 splines each with 8 small (0.4 mm^2^) closely spaced (interelectrode distance of 2.5 mm) electrodes. The length of the spindle from pole to pole was 18.4 mm with a width of 3 mm. Given the confined epicardial space, the mini‐basket catheter was in a collapsed state when used to map the epicardial surface (Figure 1). This shape also protected the epicardial coronary vasculature from the edges of the splines during catheter manipulation. In its collapsed form, the multipolar catheter was shaped like a spindle and, with its multipolar electrodes along it, maintained excellent tissue contact during catheter manipulation inside the pericardium.

The mini‐basket catheter was paired with the 3‐dimensional Rhythmia mapping system (Boston Scientific), which automatically collected chamber geometry and electrograms for voltage and timing annotation using the operator‐defined beat‐by‐beat acceptance criteria for cycle length, freedom of respiration gating, catheter stability, electrogram accuracy, tracking quality, and ECG morphology, as described previously.^6^ The filters for bipolar and unipolar electrograms were set at 30 to 300 Hz and 1 to 300 Hz, respectively.

### Substrate Mapping

**Figure 2:**
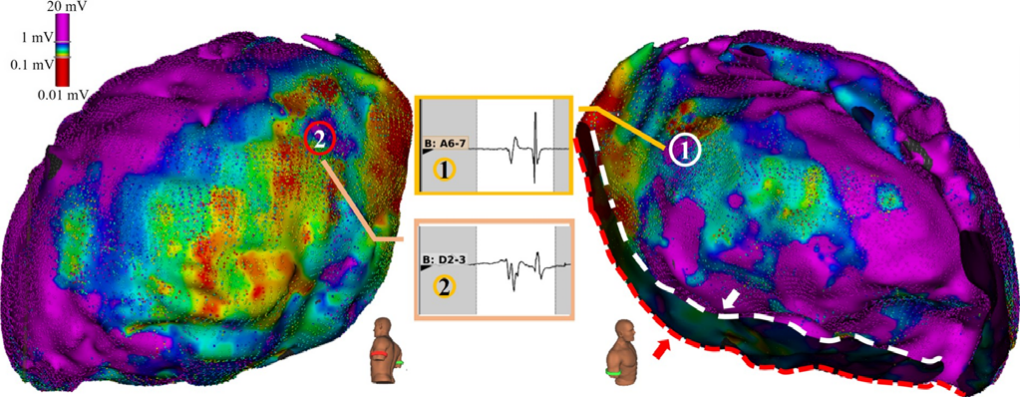
Inner and outer epicardial electroanatomical shells constructed by a mini‐basket catheter. The white arrow points to the inner surface of the epicardial substrate map (right), whereas the red arrow shows the outer surface (left). The middle panels illustrate the epicardial electrograms on the inner side (1) and counterpart outer side (2) of the epicardial space. The electrograms of the inner surface of the myocardial anatomical shell are the near‐field electrograms recorded by the catheter electrodes in contact with the epicardium, whereas the displayed outer surface is the far‐field electrogram.

The epicardial cardiac shell created by the mini‐basket catheter had an inner surface and an outer surface, as shown in Figure 2. The map on the inner surface of the myocardial anatomical shell was based on the near‐field electrogram measured from the electrodes that were in direct contact with the epicardial surface. The information displayed on the outer surface of the cardiac anatomical shell was based on the far‐field electrogram detected from the electrodes located on the opposite splines. Thus, the inner side of the acquired epicardial shell represented more clinically relevant data and was used for analysis.

The optimal bipolar voltage thresholds to identify critical sites in the epicardium with this mapping system have not been validated, although the reference value for defining abnormal bipolar electrogram voltage in the epicardium was previously established at <1.5 mV with the conventional mapping system.^1^ Preliminary reports suggest that even lower voltage cutoff values can be used to identify critical sites for VT ablation using this high‐resolution mapping system.^6^, ^12^ Thus, we applied the bipolar voltage between 0.1 to 1.0 mV as the ventricular low‐voltage scar border zone in this study.

LAVAs were defined as sharp high‐frequency fractionated electrograms during or after the far‐field ventricular electrograms in sinus rhythm or before the far‐field ventricular electrograms during VT.^11^ Using this system, the sharp high‐frequency fractionated electrograms during the ventricular systolic activation were manually tagged during the mapping procedure.^11^ The sharp high‐frequency fractionated electrograms after the ventricular systolic activation (late potentials) were automatically annotated when the timing window of interest was set after the end and before the beginning of the QRS on surface ECG.

### Activation Mapping

The mechanisms of epicardial VT based on the activation maps were classified as *focal* or *reentrant*. For the reentrant VT, an activation map was “complete” when coverage was ≥90% of the VT cycle length.

### Catheter Ablation

The earliest bipolar electrogram with a QS complex unipolar signal for focal VT and the critical isthmus demonstrated for reentrant VT were targeted during ablation using an open saline‐irrigated catheter with power delivery that ranged from 20 to 50 W, with an irrigation flow rate of 10 to 30 mL/min during ablation. The substrate‐modification‐only strategy targeting LAVA was adopted if clinical VT could not be reproducibly initiated or if VT, when induced, was hemodynamically unstable. In anatomical regions of risk, coronary angiography and phrenic nerve localization by pacing capture were performed before epicardial ablation for guidance to avoid epicardial coronary arterial or phrenic nerve injury.

Acute success of epicardial VT ablation was defined as termination of VT by ablation and/or noninducibility of clinical VT and complete elimination of LAVA for substrate modification.

### Follow‐up

The patients were closely observed for at least 48 hours after the procedure to monitor any procedure‐related acute complications, including pericardial tamponade, epicardial coronary injury, phrenic nerve injury, extracardiac organ injury, and mortality. All patients were then followed in the outpatient clinic at 3‐ and 6‐month intervals. Follow‐up data after the procedures were collected from implanted cardiac device logs and hospital admission records for recurrence of arrhythmia and other morbidity and mortality events. Recurrence of VT was defined as any sustained VT >30‐second duration detected by ambulatory cardiac monitoring devices or implanted cardiac devices with or without symptoms.

### Statistical Analysis

Data distributions were expressed as mean±SD or median (interquartile range [IQR]). Continuous variables were compared using the *t* test if data distribution met the criteria for normality; otherwise, the Wilcoxon and Mann–Whitney *U* tests were used. All analyses were performed with SPSS v18.0 (IBM Corp). *P*<0.05 was considered to be statistically significant.

## Results

### Patients

In total, 33 consecutive patients (mean age: 61±16 years; 31 male) who underwent epicardial electroanatomical mapping with the mini‐basket catheter for suspected epicardial VT were included. The baseline clinical characteristics of patients are summarized in Table 1.

**Table 1:** Clinical Characteristics of Patients

Characteristic	Result
Total patients	33 (100)
Age, y, mean±SD	61±16
Male	31 (94)
Underlying structural heart disease	
ICM	6 (18)
NICM	12 (36)
ARVC	9 (27)
HCM	2 (6)
Myocarditis	4 (12)
Syncope	13 (39)
ICD/CRT‐D implanted	28 (85)
NYHA class III/IV	13 (39)
LVEF, %	42±15
Antiarrhythmic drugs	
Class I	2 (6)
Class II	29 (88)
Class III	25 (76)
Prior VT ablation procedure	
Endocardial ablation only	17 (52)
Endocardial plus epicardial ablation	5 (15)
Prior cardiac surgical procedure	1 (3)
Reasons of epicardial procedure	
Failed endocardial ablation	22 (67)
Likelihood of epicardial VT by cardiac pathology	19 (58)
ECG suggesting epicardial source	4 (12)

Data are shown as n (%) except as noted. ARVC indicates arrhythmogenic right ventricular cardiomyopathy; CRT‐D, cardiac resynchronization therapy defibrillator; HCM, hypertrophic cardiomyopathy; ICD, implantable cardioverter‐defibrillator; ICM, ischemic cardiomyopathy; LVEF, left ventricular ejection fraction; NICM, nonischemic dilated cardiomyopathy; NYHA, New York Heart Association; VT, ventricular tachycardia.

An implantable cardioverter‐defibrillator or cardiac resynchronization therapy defibrillator was implanted in 28 patients (85%). Twenty‐four (86%) of these patients previously received appropriate therapies for VT (antitachycardia pacing, n=11; defibrillation therapy, n=21).

### Safety and Feasibility of Epicardial Mapping Using the Mini‐Basket Catheter

The mini‐basket catheter was advanced to the epicardial space via an 8.5F steerable sheath in all patients. Manipulation of the catheter in its collapsed state was straightforward. The bidirectional function of the catheter, together with the steerable sheath allowed the mini‐basket catheter to reach all aspects of the epicardial surface in all patients. There was no complication with the catheter crossing the coronary vasculature, interventricular groove, or atrioventricular groove. No hemopericardium was reported during the mapping procedure.

The epicardial apex could not be annotated by the mapping annotation system in one substrate map (1/32) and in one VT activation map (1/11) because of the catheter moving beyond the outer limits of the magnetic tracking range of the mapping system in the anterior aspect of the pericardium (Figure S1 and Discussion).

### VT Activation Mapping

Eleven epicardial activation maps were acquired during hemodynamically tolerated VT in 11 patients. The mapping data for each patient are shown in Table 2. The average VT cycle length was 441±88 ms. Five VTs (45%) had a left bundle‐branch block QRS pattern, and 6 (55%) had a right bundle‐branch block QRS pattern.

**Table 2:** Details and Characteristics of Epicardial VT Activation Mapping

Patient	Etiology	ICD/CRT‐D	Previous Ablation	Mapped Chamber	CL of VT (ms)	Mechanism of VT	Location	Epicardial Ablation	VT Termination	Inducibility of VT After Ablation
70 M	NICM	CRT‐D	Endocardial	LV	420	Focal	LV basal	Yes	Yes	No
58 M	HCM	ICD	Endocardial	LV+RV	533	Focal	RV apex	Yes	No	Yes
26 M	ARVC	No	Endocardial+epicardial	RV	410	Focal	RV lateral	Yes	Yes	No
66 M	ICM	No	No	RV	488	Reentrant	RV lateral	Yes	Yes	No
71 M	ICM	No	Endocardial+epicardial	LV	400	Focal	LV lateral	Yes	Yes	No
59 M	NICM	ICD	Endocardial	LV	521	Focal	LV anterior	Yes	Yes	No
73 M	Myocarditis	No	Endocardial	RV	304	Reentrant	RV lateral	Yes	Yes	No
76 M	ICM	No	Endocardial+epicardial	LV	485	Reentrant	LV inferolateral	Yes	Yes	No
33 M	Myocarditis	ICD	Endocardial+epicardial	LV	560	Reentrant	LV mid lateral	Yes	Yes	No
52 M	Myocarditis	ICD	No	LV+RV	373	Focal	LV lateral	Yes	Yes	No
71 M	Myocarditis	No	Endocardial	LV+RV	340	Focal	RV lateral	Yes	Yes	No

ARVC indicates arrhythmogenic right ventricular cardiomyopathy; CL, cycle length; CRT‐D, cardiac resynchronization therapy defibrillator; HCM, hypertrophic cardiomyopathy; ICD, implantable cardioverter‐defibrillator; ICM, ischemic cardiomyopathy; LV, left ventricle, NICM, nonischemic dilated cardiomyopathy; RV, right ventricle; VT, ventricular tachycardia.

**Figure 3:**
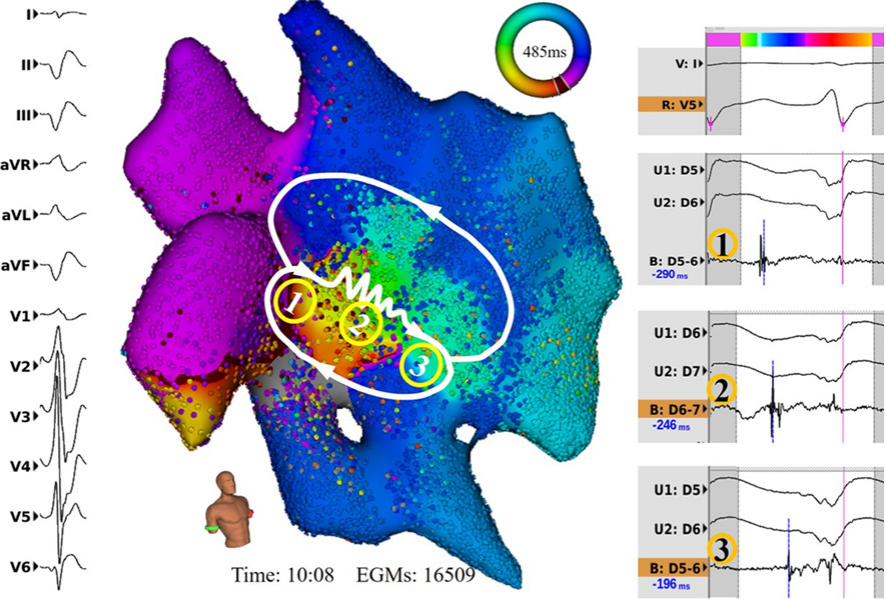
Epicardial electroanatomical maps of a macroreentrant ventricular tachycardia (VT). This 76‐year‐old male patient with ischemic cardiomyopathy underwent epicardial VT ablation due to failed previous endocardial and epicardial VT ablation. A macroreentrant VT circuit (cycle length: 485 ms) was mapped. The critical isthmus (22 mm in length and 14 mm in width) located at the left ventricle lateral epicardial wall is demonstrated from the activation map (left). Local bipolar electrograms of (1) the entrance, (2) midisthmus, and (3) the exit are shown (right).

**Figure 4:**
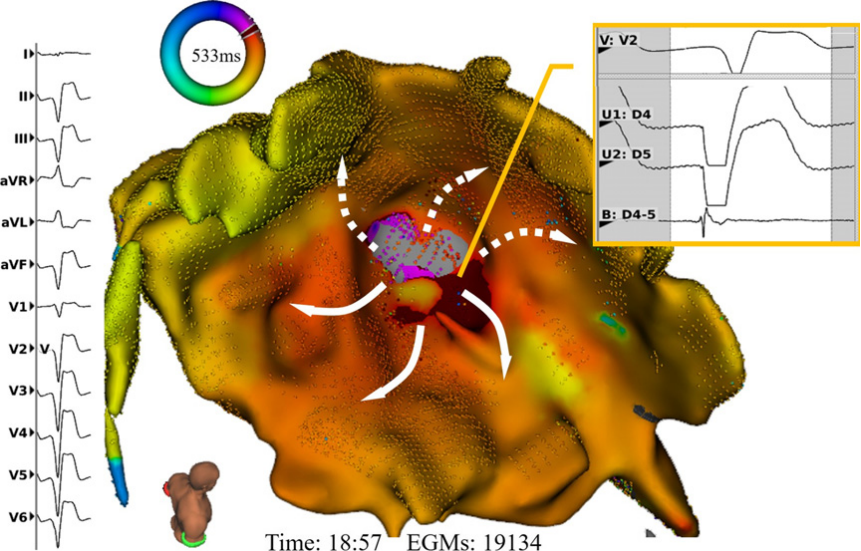
Epicardial local activation map showing focal ventricular tachycardia (VT). This 58‐year‐old male patient with hypertrophic cardiomyopathy underwent epicardial VT ablation due to failed previous endocardial ablation. The VT activation map demonstrates focal VT (cycle length: 533 ms). The earliest local bipolar electrogram was 28 ms ahead of QRS of surface ECG, with a QS complex of the unipolar signal at the breakout point in the right ventricle (RV) apex.

The timing of the bipolar electrogram was automatically annotated by the system with beat‐by‐beat QRS morphology recognition during mapping. The median acquisition time per VT activation map was 21 minutes (IQR: 9–24 minutes) with 17 832 electrogram data points (IQR: 7621–32 497 data points) per map. Of the 11 VT activation maps, 4 were found to be reentrant in mechanism. In all 4 patients, the complete mapping of the VT circuits, covering >90% of VT cycle length, were identified (Figure 3). The median dimension of the VT isthmus was 25 mm (IQR: 18–28 mm) in length and 14 mm (IQR: 14–15 mm) in width. The other 7 VT activation maps suggested a focal mechanism (Figure 4). Video S1 demonstrates the dynamic propagation map of a reentrant VT.

### Substrate Mapping

**Figure 5:**
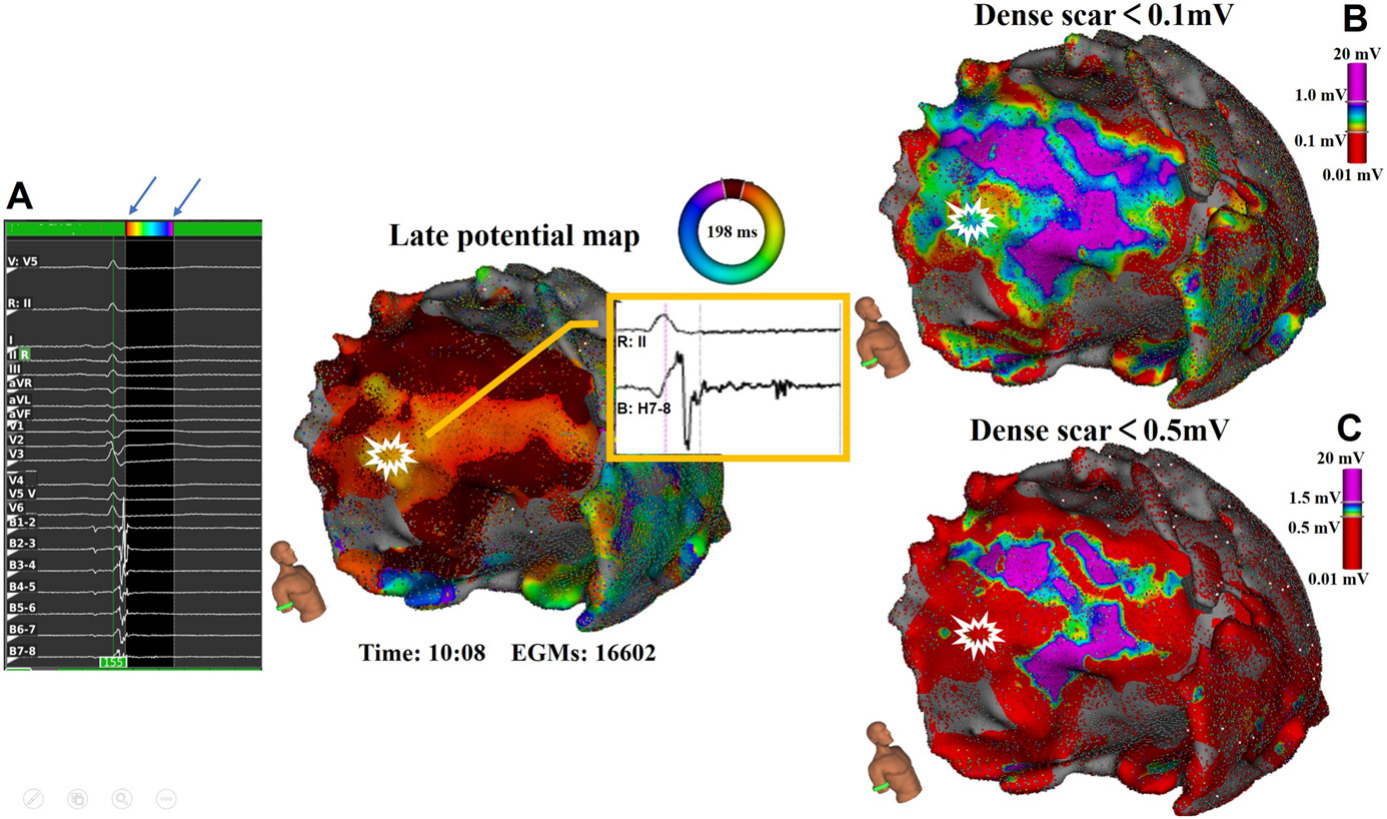
The late potential map and voltage maps annotated using different bipolar voltage thresholds. **A**, Late potential map with the reference timing window of interest set after surface QRS, and the red arrows point to electrograms with late potential; the earliest activation area of late potentials is highlighted (maroon) at the basolateral left ventricle epicardial wall. **B** and **C**, Scar border zone area delineated using bipolar voltage thresholding values of 0.1 to 1.0 mV and 0.5 to 1.5 mV, respectively, in the same patient. The late potential region (white mark) co‐correlated better with the scar border zone shown (**B**), whereas the same area was shown as scar core in (**C**).

Thirty‐two epicardial substrate maps in 30 of 33 patients were obtained. A median of 40 149 electrograms (IQR: 20 926–49 391 electrograms) were acquired per map. Epicardial scar and scar border region according to bipolar voltage threshold (dense scar <0.1 mV; scar border zone 0.1–1.0 mV) were identified on all substrate maps. Furthermore, the scar border zone delineated as 0.1 to 1.0 mV correlated better with late potential regions than that delineated as 0.5 to 1.5 mV (Figure 5).

**Figure 6:**
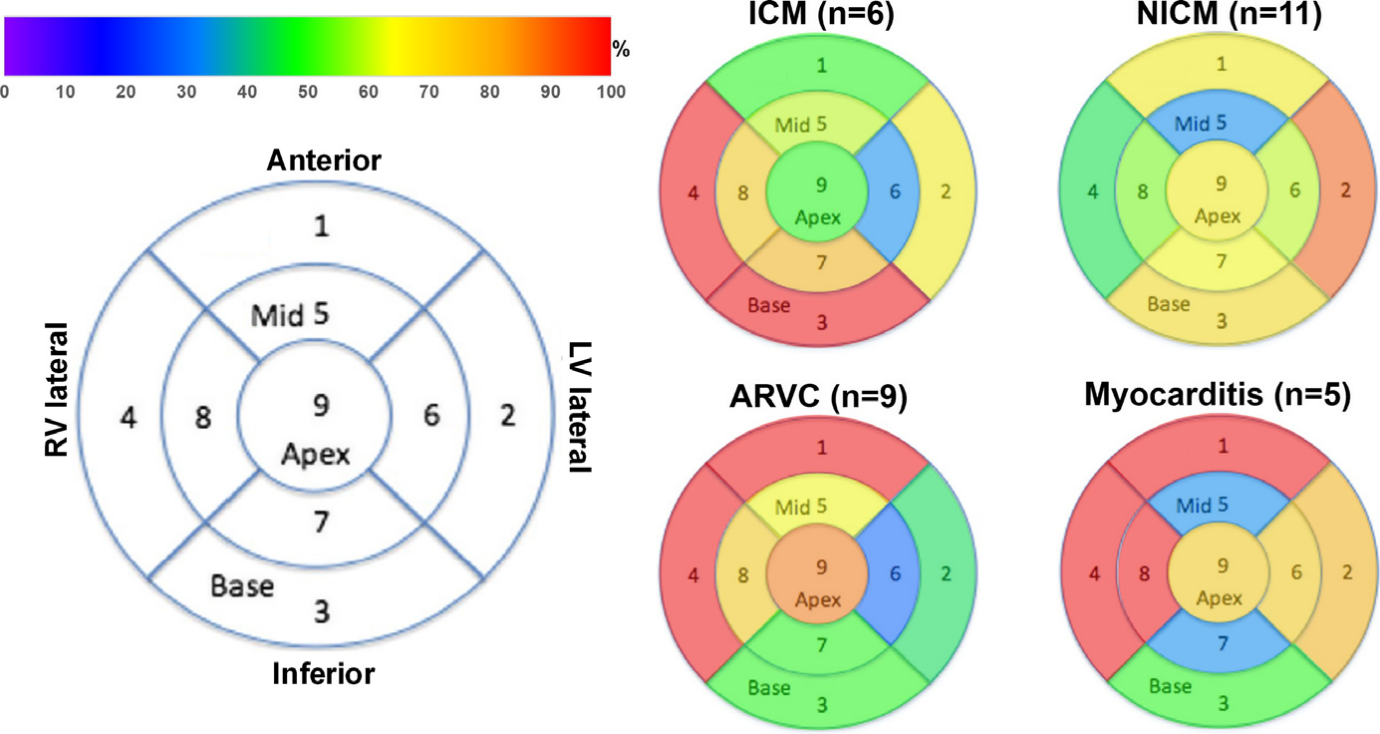
The distribution of epicardial low‐voltage areas in 4 myopathic etiologies. The polar diagram (left) demonstrates the anatomical segmentation of the epicardial ventricular wall. The color bar demonstrates the percentage of low‐voltage area (<1.0 mV) observed in each segment. The 4 polar charts (right) demonstrate the distribution of scar locations by the percentage of patients with each respective etiology. ARVC indicates arrhythmogenic right ventricular cardiomyopathy; ICM, ischemic cardiomyopathy; LV, left ventricle; NICM, nonischemic dilated cardiomyopathy; RV, right ventricle.

The distribution of epicardial low‐voltage areas categorized by cardiac pathology is shown in Figure 6. The most common epicardial low‐voltage distribution for the ischemic cardiomyopathy cases was at the basal right ventricular (RV) lateral and left ventricular (LV)/RV inferior wall; for nonischemic dilated cardiomyopathy, it was at the LV basal lateral wall; for arrhythmogenic RV cardiomyopathy, it was at the basal RV lateral and RV/LV anterior wall; and for myocarditis, it was at the basal to mid‐RV lateral wall and the basal LV/RV anterior wall. For the one available substrate map in a patient with hypertrophic cardiomyopathy, low‐voltage areas were widespread across the all epicardial walls.

The median areas identified as dense scar and scar border zone were 7.7 cm^2^ and 30.6 cm^2^, respectively, for patients with ischemic cardiomyopathy; 3.6 cm^2^ and 27.7 cm^2^, respectively, for patients with nonischemic dilated cardiomyopathy; 8.2 cm^2^ and 40.0 cm^2^, respectively, for patients with arrhythmogenic RV cardiomyopathy; and 3.6 cm^2^ and 26.7 cm^2^, respectively, for patients with myocarditis. There was no significant difference in the size of dense scar (*P*=0.227) and border zone (*P*=0.464) between the groups.

In 30 of 32 epicardial substrate maps, LAVA was recorded and its location co‐correlated with the scar border zone. A simulated “roving probe” allowed immediate review of each point in regions of interest to confirm and define the areas of LAVA during the procedure.

### Complications

There were no immediate procedural complications. Two delayed pericardial collections required reinsertion of the pericardial drain 1 day after the procedure, with no long‐term sequelae. Both patients received anticoagulation for simultaneous endocardial mapping, and both drains were removed immediately after the procedure. There was no incidence of cardiac tamponade, phrenic nerve injury, or coronary vascular injury during the electroanatomical mapping procedure. There was no report of delayed clinically significant pericarditis.

### Acute and Medium Outcomes

In total, 31 of 33 patients who had epicardial electroanatomical mapping with the mini‐basket catheter underwent epicardial ablation. Two patients did not have epicardial ablation because the maps indicated that the VT exit was endocardial, following additional endocardial mapping. The median total ablation time was 25 minutes (IQR: 15–33 minutes) with a median of 20 energy applications (IQR: 10–30 applications).

The patients with activation maps underwent epicardial ablation targeting either the isthmus or the earliest activation point, with successful termination of 10 of 11 VTs during radiofrequency delivery. In 1 of 11 patients, VT could not be terminated epicardially but was terminated with additional endocardial ablation. Additional substrate modification after VT termination was performed in 8 of 11 patients. None had inducible VT after the ablation.

In the 20 patients who underwent a substrate‐modification‐only strategy, 3 patients had combined endocardial and epicardial ablation of LAVA. Complete elimination of LAVA was achieved in 17 of 20 patients, and partial elimination was achieved in 3 of 20 patients. The reason for partial LAVA elimination in the 3 patients was to avoid coronary vessel or phrenic nerve injury.

At a median follow‐up of 10 months (IQR: 4–14 months), freedom from VT was 64% (7/11) in those patients who had acute termination of VT during radiofrequency delivery plus additional substrate modification and 55% (11/20) in those who had substrate modification only. The recurrence of VT occurred within a median duration of 6 months (IQR: 3–9 months) after the procedure, with new VT morphology documented in 60% of recurrences. There were 3 deaths, at 1, 2, and 12 months after the respective ablation procedures, due to intractable heart failure while on the waiting list for heart transplantation.

## Discussion

This study is the first to report on the clinical utility of a 64‐pole mini‐basket catheter to map epicardial VT at 5 large‐volume centers. The main findings are that this high‐density mapping technology is able to identify all reentry circuits, the earliest activation foci, and ventricular substrate to guide epicardial ablation treatment of VT with favorable immediate and medium‐term ablation outcome; in a collapsed state, the mini‐basket catheter is safe to maneuver within the confined space of the pericardium without immediate complication from the mapping procedure. The longer term clinical outcome from using this rapid, high‐density, and high‐resolution electroanatomical mapping to guide epicardial ablation procedures will have to be assessed in further larger studies.

### Safety and Feasibility

The body‐surface ECG morphology of VT and the etiology of the cardiac pathology may indicate the epicardial origin of VT, but ultimately it requires confirmative and detailed epicardial electroanatomical mapping. A combined endoepicardial approach is becoming the first‐line approach for catheter ablation treatment of suspected epicardial VT. Our prior reports have demonstrated the merits of the novel multipolar mini‐basket catheter in endocardial mapping and ablation of a variety of arrhythmias.^6^, ^7^, ^13^ Its application in the epicardial space, however, was outside the “instruction of use” of the mini‐basket catheter and has been reported in only a single case report.^14^ The present study showed that when the multipolar catheter was used in its collapsed state, forming a spindle shape as opposed to the mini‐basket, it moved freely within the pericardial space while maintaining excellent contact with the epicardium. The edges of each spline were tightly opposed to each other, minimizing the perceived risk of injuring the coronary vasculature within the pericardial space. Furthermore, the splines of the catheter were soft and could be easily manipulated in the limited space. All anatomical regions were reached with the assistance of the steerable sheath.

There was no reported hemopericardial collection or significant bleeding during the mapping part of the procedure. The 2 cases of delayed pericardial effusion after the removal of the drain following the procedure were treated successfully by reinsertion of the drain. There was no reported clinically significant late pericardial inflammatory process in this cohort. Overall, the potential rate of complications associated with epicardial VT catheter mapping using the mini‐basket catheter in this cohort was relatively similar to that of the previous report using the conventional mapping catheter and system.^3^, ^15^

### Accuracy of Electroanatomical Mapping

Anter et al previously showed that catheters with small and closely spaced electrodes can better detect viable myocardial tissue in zones of low‐voltage tissue and record near‐field larger bipolar voltage amplitude in comparison with catheters with larger electrodes and greater interelectrode spacing.^5^ The multipolar mini‐basket catheter with its small electrodes coupled with the close interelectrode spacing arrangement, especially in its closed state in the pericardial space, provided not only high‐density but also high‐resolution electroanatomical mapping with clear demonstration of LAVAs and low amplitude mid‐diastolic potentials. Even when the catheter is in its collapsed state, the flexibility of the spindle and the close spacing of the electrodes along each spline allowed at least 2 to 3 splines in direct contact with the epicardial myocardial surface at any time.

In conjunction with its specific algorithm for point acquisition and annotation, rapid high‐density maps were acquired with high accuracy. In this study, all reentrant VT isthmuses were clearly identified in the epicardium. As one would expect, the isthmus size is larger in human (25 mm in length and 14 in width) than in the infarcted swine model (16 mm in length and 7 mm in width) by high‐density mapping.^16^ In this small cohort, focal VT was perhaps more prevalent than expected (7/11). The site of earliest epicardial activation was localized in all patients, with successful termination by epicardial ablation in 6 of 7 patients and endocardial ablation in the remaining 1 patient. This higher prevalence of focal VT is a reflection of the patient cohort with nonischemic pathology and mappable VT. However, an intramural or transmural reentry circuit with epicardial exit is also possible.^17^, ^18^

The epicardial substrate demonstrated by the multipolar mini‐basket catheter paired with the 3‐dimensional mapping system showed that the most common low‐voltage regions in the patient with nonischemic dilated cardiomyopathy were distributed on the LV lateral wall, with arrhythmogenic RV cardiomyopathy on RV lateral wall, and with myocarditis on the RV lateral wall and near the annulus. This finding is consistent with the results of prior studies.^1^, ^19^, ^20^, ^21^ It is worth noting that epicardial fat may still be a confounder of perceived scar region despite the use of such catheter technology.^22^

### Automated Annotation of Electrograms

The automated annotation function of this 3‐dimensional mapping system is accurate overall. Nonetheless, the automated algorithm ought to be used with caution when annotating the diastolic or late potentials. The algorithm is designed to annotate the largest amplitude electrogram at the earliest dV/dt within a predefined mapping window or period. Therefore, at those sites where there are 2 potentials within the mapping window (a systolic potential plus a diastolic potential in VT or late potential in sinus/paced rhythm), the automated function may not annotate the clinically relevant potential, which is often smaller than that for systolic ventricular activation. Under such circumstances, manual adjustment of the mapping window may be necessary to blank the systolic duration when large systolic ventricular electrograms reside.^5^ Annotation of complex and fractionated electrograms like LAVA, that is, within or close to the ventricular systolic activation, will have to be done manually with the current version of software.

### Mapping Field

The outer limit of the magnetic tracking was reached in 2 patients when mapping in the anterior pericardial space. This limit varies between different procedure laboratories given the distance between the magnetic generator and the patient's anterior cardiac silhouette, different tables, mattress thickness, and baseline magnetic interference. Users should anticipate this limitation if the patient is large with a barrel‐shaped chest. Furthermore, the “Tracking and Motion” function may need to be disabled when mapping in the epicardial space. The reason is that the tracking metric for the multipolar catheter is based on the magnetic field but also on the deployment of the mini‐basket catheter. The degree of deployment is measured by the voltage change among 4 electrodes of the mini‐basket catheter. In the epicardial space, inconsistent fluid volume among the 4 electrodes may affect this; therefore, when the tracking metric is uncertain, the motion function cannot be used.

### Clinical Outcome

In this study group, the epicardial VT reentry circuits and the earliest activating foci could be clearly identified when the tachycardia was inducible and hemodynamically tolerated. Eleven inducible VTs were accurately mapped, and ablation at the identified isthmus and earliest activation sites led to the termination of VT in 10 of 11 patients by epicardial ablation during VT. The termination rate during epicardial ablation was 91% (10/11), with 64% free from VT at a median of 10 months of follow‐up. In addition to termination of VT and noninducibility of clinical VT, substrate LAVA elimination has been shown to be an end point predicting medium‐ and long‐term success of the procedure.^11^ In 20 patients who had epicardial substrate modification only, complete LAVA elimination was achieved in 17 of 20 patients (85%) and related to 55% freedom from VT. This finding is comparable to the results of prior studies.^3^, ^4^

### Limitation

This was a nonrandomized prospective observational study that carried the inherent shortcomings of such a study design. Nevertheless, it was the largest cohort of epicardial VT cases utilizing the mini‐basket catheter paired with a 3‐dimensional mapping system from the collective experiences of 5 high‐volume European centers. There was no validation of the electroanatomical data with imaging or histological data that would correlate with the size and location of the scar and border zone. The patient cohort was heterogeneous; however, it consisted of consecutive patients with a variety of common cardiac pathologies, reflecting real‐world experiences.

## Conclusion

Epicardial mapping with the 64‐pole multipolar mini‐basket catheter in its collapsed state, paired with the automatic high‐density mapping system, is safe and feasible for identifying all reentry circuits, the earliest activation foci, and ventricular substrate to guide epicardial VT ablation. Further large‐scale prospective studies including long‐term follow‐up data are warranted to determine a potential clinical benefit.

## Disclosures

Frederic Sacher and Vias Markides have received speaking honorarium from Boston scientific (modest). Rui Shi has received a fellowship scholarship from the China Scholarship Council (modest). The remaining authors have no disclosures to report.

## Supporting information


**Video S1.** The dynamic propagation map of a macroreentrant epicardial VT. The propagation map suggested the presence of a critical isthmus located at the LV lateral epicardial wall.
