# Supplementary material for: Epicardial Ventricular Tachycardia Ablation Guided by a Novel High‐Resolution Contact Mapping System: A Multicenter Study
Source: J Am Heart Assoc. 2018 Oct 30;7(21):e010549. doi: 10.1161/JAHA.118.010549 (PMC6404200; doi:10.1161/JAHA.118.010549)
Supplement: Supplementary file 2 [file JAH3-7-e010549-s002.pdf]

SUPPLEMENTAL MATERIAL

Video S1. The dynamic propagation map of a macroreentrant epicardial VT. The propagation map suggested the presence of a critical isthmus located at the LV lateral epicardial wall.
